# A method to reconstruct and apply 3D primary fluence for treatment delivery verification

**DOI:** 10.1002/acm2.12017

**Published:** 2016-12-08

**Authors:** Shi Liu, Thomas R. Mazur, Harold Li, Austen Curcuru, Olga L. Green, Baozhou Sun, Sasa Mutic, Deshan Yang

**Affiliations:** ^1^ Department of Radiation Oncology School of Medicine Washington University St. Louis MO USA

**Keywords:** IMRT, quality assurance, radiation therapy, VMAT

## Abstract

**Motivation:**

In this study, a method is reported to perform IMRT and VMAT treatment delivery verification using 3D volumetric primary beam fluences reconstructed directly from planned beam parameters and treatment delivery records. The goals of this paper are to demonstrate that 1) 3D beam fluences can be reconstructed efficiently, 2) quality assurance (QA) based on the reconstructed 3D fluences is capable of detecting additional treatment delivery errors, particularly for VMAT plans, beyond those identifiable by other existing treatment delivery verification methods, and 3) QA results based on 3D fluence calculation (3DFC) are correlated with QA results based on physical phantom measurements and radiation dose recalculations.

**Methods:**

Using beam parameters extracted from DICOM plan files and treatment delivery log files, 3D volumetric primary fluences are reconstructed by forward‐projecting the beam apertures, defined by the MLC leaf positions and modulated by beam MU values, at all gantry angles using first‐order ray tracing. Treatment delivery verifications are performed by comparing 3D fluences reconstructed using beam parameters in delivery log files against those reconstructed from treatment plans. Passing rates are then determined using both voxel intensity differences and a 3D gamma analysis. QA sensitivity to various sources of errors is defined as the observed differences in passing rates. Correlations between passing rates obtained from QA derived from both 3D fluence calculations and physical measurements are investigated prospectively using 20 clinical treatment plans with artificially introduced machine delivery errors.

**Results:**

Studies with artificially introduced errors show that common treatment delivery problems including gantry angle errors, MU errors, jaw position errors, collimator rotation errors, and MLC leaf position errors were detectable at less than normal machine tolerances. The reported 3DFC QA method has greater sensitivity than measurement‐based QA methods. Statistical analysis‐based Spearman's correlations shows that the 3DFC QA passing rates are significantly correlated with passing rates of physical phantom measurement‐based QA methods.

**Conclusion:**

Among measurement‐less treatment delivery verification methods, the reported 3DFC method is less demanding than those based on full dose re‐calculations, and more comprehensive than those that solely checks beam parameters in treatment log files. With QA passing rates correlating to measurement‐based passing rates, the 3DFC QA results could be useful for complementing the physical phantom measurements, or verifying treatment deliveries when physical measurements are not available. For the past 4+ years, the reported method has been implemented at authors’ institution 1) as a complementary metric to physical phantom measurements for pretreatment, patient‐specific QA of IMRT and VMAT plans, and 2) as an important part of the log file‐based automated verification of daily patient treatment deliveries. It has been demonstrated to be useful in catching both treatment plan data transfer errors and treatment delivery problems.

## 1 Introduction

In intensity‐modulated radiotherapy (IMRT)^1^ and volumetric‐modulated arc therapy (VMAT),^2^ radiation is delivered in many individual beam apertures of varying intensities to achieve highly conformal dose distributions to the planning target volume (PTV), that minimize dose to nearby health tissues.^3^ During delivery, mechanical parameters (e.g., MU, dose rate, gantry angle, collimator angle, jaw position and MLC leaf positions) are synchronized to planned values, specified by control points (CP).^4^ Given the complexity of these treatments, quality assurance (QA) for treatment delivery is essential in detecting various types of delivery failures in order to ensure the accuracy of a patient's dosimetry and safety. IMRT/VMAT QA can be performed using point dose and planar dose measurements obtained via physical phantoms, 2D beam fluences, and dose recalculations based on machine delivery log files.^4^, ^5^, ^6^ In comparison to conventional measurement‐based QA, QA using log files offers various advantages including sampling higher spatial and temporal resolutions, not requiring measurement devices or phantoms, providing QA for fractional deliveries to patients, and being readily automated.^5^, ^6^ Performing IMRT QA using log files has been claimed to be more effective and efficient than, and complementary to, physical dose measurement‐based QA.^7^, ^8^, ^9^, ^10^, ^11^, ^12^ A major, ongoing debate in the medical physics community is whether IMRT QA using log files can replace conventional measurement‐based methods.^5^

Numerous reports on using log files for IMRT/VMAT QA have been presented in literature.^13^, ^14^, ^15^, ^16^, ^17^, ^18^ Logged beam parameters can be compared to planned values based on a relatively simple value‐to‐value comparison. Dose recalculations that incorporate parameters recorded in log files can verify the accuracy of delivered dose. Computation time has been significantly reduced with GPU acceleration;^19^, ^20^, ^21^, ^22^, ^23^ however, comparing dose distributions can be complicated by differences in dose calculation engines and treatment planning systems (TPS), the accuracy of electron density determined in the daily patient localization cone‐beam CT images, and other factors.

Traditionally in IMRT QA, verification of delivered 2D fluence maps for individual beams has been widely used.^8^ Two‐dimensional beam fluence can be directly measured with various dosimeters including diode or ion chamber arrays, e.g., MapCheck (Sun Nuclear, Melbourne, FL, USA) or MatriXX (IBA, Bartlett, TN, USA), or even with onboard electronic portal imaging devices (EPID).^4^, ^24^, ^25^ Beam 2D fluence can also be digitally and proximately reconstructed from treatment plan parameters or LINAC machine log files by integrating across a beam aperture multiplied by the per‐segment beam MU.^10^, ^26^, ^27^, ^28^ At the authors’ institution, 2D beam fluences digitally reconstructed from the DICOM plans and treatment delivery log files have enabled detection of many errors for IMRT plans, including human operating mistakes (resulting in wrong plans, wrong beams, or wrong beam parameters), flawed and suboptimal treatment plans (containing undeliverable or incorrect machine parameters), data transfer problems (resulting from unintended parameter changes), and other minor false positive errors.^11^ However, for the case of VMAT QA, such 2D beam fluence verification per beam angle may not be appropriate because instantaneous beam aperture errors for VMAT deliveries were significant (up to 15%) for highly modulated plans even though MLC leaves were well‐within tolerances.^29^ A composite 2D fluence for a VMAT beam at a fixed gantry angle could be computed,^30^ but error detection using such fluence is suboptimal due to the ignored gantry rotation. We therefore were motivated to develop an alternative 3D fluence calculation QA method, i.e., 3DFC, that (1) could be more sensitive to detect certain delivery machine errors (such as gantry rotation errors), (2) could provide enhanced visualization of beam delivery discrepancies respective to the tumor target geometry, and (3) could infer correlations between random treatment delivery discrepancies to dose discrepancies.

In this study, a simple and efficient QA method based on 3D fluence calculation is reported. This method enables rapid calculation of 3D fluences using beam parameters from machine log files and DICOM plan files. Our goal is not to replace traditional physical phantom measurement‐based QA or a full‐scale dose calculation, but rather to present a simpler, complimentary solution for detecting potential delivery machine parameters errors and plan parameter transfer errors with improved 3D visualization. The reported 3DFC QA method mainly focuses on checking delivery errors of machine parameters — instead of scrutinizing TPS commissioning errors — while potentially improving error sensitivities comparing to the traditional QA methods. Toward this goal, we examine correlations between the resultant passing rates from our reported 3DFC QA and conventional measurement‐based QA in detail.

## 2 Materials and methods

### 2.1 Data

To calculate and verify the 3D fluence volume, both the planned beam parameters from DICOM plans and the reported beam parameters from the beam delivery log files are used. Beam parameters are defined similarly in the DICOM plans and machine log files. In DICOM plans, beam parameters are defined in control points, which are checkpoints for the treatment machines to synchronize beam parameters. For example, a control point defines the gantry, collimator, jaw, and MLC leaf positions, as well as the accumulated beam monitor units (MU) up to this control point. In machine log files, each record stores the same beam and dosimetric parameters measured at fixed intervals throughout delivery. The currently supported machine log files are pre‐TrueBeam dynamic MLC log files (dynalog) and TrueBeam trajectory log files, both of which are acquired on Varian linear accelerators. Records are generated every 50 and 20 milliseconds for pre‐TrueBeam and TrueBeam machines, respectively. TrueBeam trajectory logs also give absolute beam MUs and dose rate, while dynalog files only give relative beam MU values.^31^ To be concise, only the TrueBeam trajectory log files and VMAT plans will be discussed in the following sections.

### 2.2 3D fluence calculation (3DFC)

Two‐dimensional beam fluence can be digitally computed based from machine log files by integrating the per‐segment beam aperture multiplied by the per‐segment beam MU.^10^ In contrast, the 3D volumetric fluence is calculated by forward‐projecting beam apertures, modulated by beam monitor units (MU), at all beam angles. In this paper, 2D and 3D fluence calculation methods are referred to as 2DFC and 3DFC, respectively, and IMRT and VMAT delivery QA using 2DFC and 3DFC methods are referred to as 2DFC QA and 3DFC QA, respectively.

Consider a point $\vec{r}\in \Omega$, where Ω is the target 3D fluence volume around the beam isocenter. *x*,* y* and *z* represent coordinates of the point $\vec{r}$ with the origin defined at the beam isocenter, the 3D fluence intensity $I(\vec{r})$ is calculated, using the beam parameters in the machine log files, as:

(1) $$I\left(\vec{r}\right)=\int F\left({\vec{r}}^{\prime }\left(t\right)\right)\times M\left({\vec{r}}^{\prime }\left(t\right)\right)\times \dot{D}\left(t\right)\times \frac{SAD^{2}}{|\vec{r}-\vec{s}\left(t\right)|}dt$$

where *t* is the delivery time, *F* is the 2D beam intensity profile in air, $\dot{D}$ is the dose rate in MU/s, *SAD* is the source‐to‐axis distance, $\vec{s}\left(t\right)$ is the source position, and *M* is the beam aperture mask with *M* = 1 if $\vec{r}{\;}^{\prime }$ is inside the beam aperture or *M* = 0 otherwise. $I(\vec{r})$ represents the total MU delivered to the point $\vec{r}$ by the cumulative beam aperture the entire beam delivery. It is important to note that beam attenuation and scattering are not considered as opposed to the dose calculation in this simple approximation. The computed 3D fluence is essentially the dose in air. X‐ray generated is approximated from the single radiation source at the X‐ray target, and the secondary effective source is not considered. As the mask is not binary in reality, but a function of the aperture size, the fluence for smaller apertures is reduced due to the shadowing of the distributed secondary source by the MLC. Therefore, we note that an approximation to the real mask counterpart is applied in this calculation.

$\vec{r}{\;}^{\prime }$ is the point $\vec{r}$ projected on the beam portal at 100 cm SAD and couch, gantry and collimator are all at 0°:

(2) $$\vec{r}{\;}^{\prime }\left(u,w\right)=R_{col}\left(\theta \right)P\left[R_{g}\left(-\beta \right)R_{couch}\left(-\alpha \right)\vec{r}\right]$$

where *R*_couch_, *R*_g_, and *R*_col_ are the couch, gantry, and collimator rotation matrices, respectively, and *α*,* β*, and *θ* are the beam couch, gantry, and collimator angles, respectively. *P* is a 3D‐to‐2D projection operator that projects a 3D coordinate $\vec{r}\left(x,y,z\right)$ to a point $\vec{r}{\;}^{\prime }\left(u,w\right)$ within the beam portal according to:

(3) $$\vec{r}{\;}^{\prime }\left(u,w\right)=P\left[\vec{r}\left(x,y,z\right)\right]=\left[\begin{array}{c} \frac{x\times SAD}{y+SAD}\\ \frac{z\times SAD}{y+SAD} \end{array}\right]$$

where *u* is oriented along the direction of the X‐jaws (or MLC motion), and *w* is given along the direction of the Y‐jaws.

The beam aperture mask *M* is directly calculated using the jaw and MLC leaf position data. For the projected point $\vec{r}{\;}^{\prime }$ on the beam portal at (*u, w*), the corresponding leaf pair number can be calculated using *w*. The calculation is different for different machine configurations. For a Varian Millennium 120 MLC module that has 60 MLC leaf pairs, the leaf widths are 1 cm for the first 10 and last 10 leaves, and 0.5 cm for the middle 40 leaves. Leaf pair number *L*_num_ is calculated from *w* as:

(4) $$L_{num}=f\left(int\left(\frac{w+20}{0.5}\right)+1\right)$$

Where

(5) $$f\left(\upsilon \right)=\left\{\begin{array}{cc} int((\upsilon -1)/2)+1 & \upsilon \in [1,20]\\ \upsilon -10 & \upsilon \in [21,60]\\ int((\upsilon -61)/2)+51 & \upsilon \in [61,80] \end{array}\right.$$

where “*int*” denotes the integer conversion operation. The point $\vec{r}$ is considered to be in the beam aperture if *u* is between the two leaf positions for the relevant leaf pair *L*_num_ and within the beam opening of the X and Y jaws.

For a DICOM plan, the 3D fluence is calculated similarly as:

(6) $$I\left(\vec{r}\right)=\sum_{k=1}^{N-1}\left\{F\left(\vec{r}{\;}^{\prime }\left(t\right)\right)\times M_{k}\left(\vec{r}{\;}^{\prime }\left(t\right)\right)\times \Delta MU_{k}\times \frac{SAD^{2}}{|\vec{r}-\vec{s}\left(t\right)|}\right\}$$

where *k* is the control point index and Δ*MU*_k_ is the beam MU allocated between control points *k* and *k*+1. The rotation angles used in the calculation of $\vec{r}{\;}^{\prime }$ are the averaged values between points *k* and *k* + 1. Likewise, the planned source position $\vec{s}$ is averaged between points *k* and *k* + 1 as:

(7) $$\vec{S}=\left(\vec{S_{k}}+{\vec{S}}_{k}+1\right)/2$$

### 2.3 Implementation details

The number of control points in VMAT plans is usually far less than the number of records in the machine logs. A single 360° arc with a total of 91 control points (4° per control point) within its associated plan will be delivered in 2 min. Over this duration over 5000 log records will be generated. With an angular sampling frequency of 4° per beam in the plan, the reconstructed 3D fluence volume will have apparent alias; however, the delivery machine linearly interpolates the beam parameters between control points in order to smooth the expected delivered 3D fluence. To calculate the 3D fluence with high accuracy, the control points in the DICOM plans thus need to be up‐sampled accordingly.^17^ It was empirically determined that 1° per control point sufficiently reduces alias artifacts.

On the other hand, Varian TrueBeam machines create delivery records every 20 ms with an equivalent angular resolution of 0.048°. Because such high angular resolution is not necessary for detecting gross delivery errors, machine logs are down‐sampled by a factor of 16 to improve computation speed. To combine multiple records into 1 segment, the MLC leaf positions and gantry angles are averaged and the beams MUs are summed.

The reconstruction volumes are automatically determined using the maximal jaw opening from the treatment plan (plus a 1 cm margin) given that the jaw positions are not changing during VMAT delivery. A voxel size of 3 × 3 × 3 mm^3^ and 1 degree angular resolution are used in this study in order to provide adequate spatial resolution for error detection with high fidelity and reasonable computation time.

### 2.4 3DFC QA for treatment delivery verification

**Figure 1:**
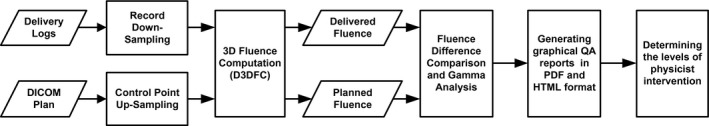
The general workflow of the VMAT delivery QA.

Figure 1 presents the general workflow of VMAT and IMRT 3DFC QA, which can be described in details as follows:

1. Obtaining the treatment plan and the machine delivery logs.
2. Calculating the planned and delivered 3D fluences from the DICOM plan delivery logs using the 3D fluence calculation method.
3. Performing an intensity difference test (3%) and gamma analysis (3%, 3 mm) between the planned and delivered fluence values^32^, ^33^, ^34^, ^35^ and computing the failing rates of both criteria, respectively.
4. Generating QA reports for physicists’ analysis and approval.
5. Intervening based on failing rates (according to the discretion of a physicist).

A 3% intensity difference and 3%, 3 mm gamma criterion^32^, ^33^, ^34^, ^35^ are chosen for defining passing rates based on 3DFC comparisons. In the 3% fluence difference test, each voxel in the planned fluence map is considered to have passed if the fluence difference between planned and delivered values on the voxel is less than 3% of the maximal intensity value. Voxels with intensity values smaller than 10% of the maximal value are excluded from analysis. The failing rates of both tests summarize the total number of voxels that fail the corresponding criterion out of the total number of voxels in the fluence map. The chosen parameters for the criteria (3% for fluence difference and 3%, 3 mm in the gamma analysis) are selected empirically in order to avoid excessive false positives while preserving sufficient sensitivity to catch major delivery errors.

Interventions of medical physicists are decided according to the estimated failing rates of the two criteria. For instance, for a lung cancer patient with up to 2 mm in simulated random MLC errors, the failing rate of the 3% fluence difference test was calculated to be 4.8%, and the failing rate in gamma analysis (3%, 3mm) was 4.1%. For a heart patient with up to 1° random gantry angle errors, the failing rate of the fluence difference test was calculated to be 2.4%, and the failing rate in gamma analysis was 0.8%. While the threshold values for the action levels should be determined with further clinical measurements and judgments, which could be treatment site dependent, the general threshold used in the authors’ clinic is 5% failing rate on 3%, 3 mm gamma analysis. If the failing rate of gamma analysis is greater than 5%, medical physicists should initiate further investigation.

### 2.5 Testing with simulated delivery errors

In order to evaluate the capabilities of the 3DFC QA to detect delivery problems, we simulate five types of important machine parameter errors by modifying the treatment plans (easier to modify than log files) and comparing the detection results with both 2DFC QA methods and conventional measurement‐based QA using ArcCHECK (Sun Nuclear Corp, Melbourne, FL, USA). We use the original unmodified treatment plan to deliver the VMAT beams such that the machine log file is recorded from the original plan delivery. Assuming the modified plan is the correct plan, errors within the fluence volume will be identified between the delivery log (from the original unmodified plan) and modified plan (with simulated errors). Testing of the simulated delivery errors includes two steps:

1. Simulation of gantry rotation errors, MU errors, jaw positions errors, collimator rotation errors, and MLC leaf errors by adding random values to the corresponding planned quantities per control point. These random artificial errors are specified according to their corresponding statistical distributions and range well within the corresponding error tolerances for the associated parameters (as enumerated in Table 1).
2. Simulation of the errors enumerated above beyond their tolerances, including adding random values between 1° and 2° per control point to both the planned gantry rotation angles and collimator rotation angles, adding random values between 1 and 2 MU per control point to planned MU values, adding random values between 1 mm and 2 mm to planned jaw positions, and shifting random values between 2 mm and 3 mm to either direction of the MLC leaf positions.

**Table 1:** Normal tolerances and statistical distributions for various sources of errors of the Varian LINAC machines installed in our clinic

Errors	Gantry	MU	Jaw	Collimator	MLC
Tolerance	1°	1 MU	1 mm	1°	2 mm
Distribution	Uniformly	Uniformly	Uniformly	Uniformly	Gaussian

The tolerances of beam delivery parameters listed in Table 1 are chosen based on the AAPM Task Group 142 report.^36^ Furthermore, the statistical distributions of these types of errors are chosen based on both the uncertainties of our machines and study reported in Ref.^18^ Our goal is to test if the 3DFC is useful to detect errors that are otherwise undetectable by value‐to‐value comparisons of beam parameters, and to also investigate our method's performance in identifying errors beyond normal tolerances. For each VMAT plan, the types of errors listed above are added one‐by‐one to the original plan. A total of 10 error‐introduced plans are thus created with five incorporating errors within tolerance and five simulating errors out of tolerance. The calculated fluences of all plans will then be compared with the delivery log file recorded from the original, unmodified plan delivery. The passing rates of the 3% intensity difference test and 3%, 3 mm gamma analysis are used to evaluate the detection performance (sensitivity) of the 3DFC QA for each type of delivery error. Then, the QA results will be compared with the performance of the 2DFC QA (with the composite 2D fluence being computed by fixing the gantry angle at 0°), and with the performance of the conventional measurement‐based QA using ArcCHECK.

### 2.6 Correlation study design

**Figure 2:**
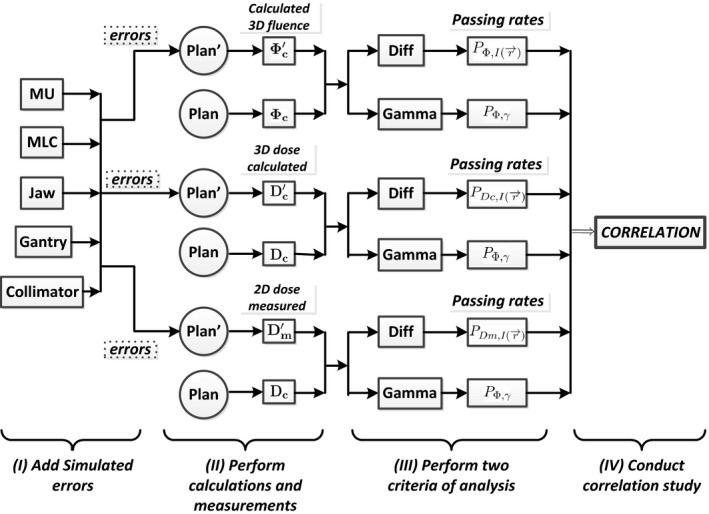
The diagram of the correlation study design between the 3DFC QA and the measurement‐based QA.

In order to evaluate the capabilities of the 3DFC QA to detect dose delivery errors, we quantitatively study the correlation between passing rates derived from fluence maps and those observed on dose measurements. Figure 2 schematically illustrates our correlation study design. Adding the same types of simulated errors to the DICOM plan (presented in Section 2.5), within their corresponding error tolerances, we calculate 3D fluences, calculate 3D doses within the Pinnacle TPS (Phillips, Pinnacle), and measure 2D composite doses using ArcCHECK, for both the original and modified plans. For each set of calculated fluences, calculated doses, and measured doses, we perform both an intensity difference test and gamma analysis test in order to evaluate the differences between the original plan and the error‐introduced plans. Finally, a correlation study is conducted on the resultant passing rates, between the fluence and the calculated dose, and between the fluence and the measured dose.

The notation shown in Fig. 2 can be summarized as follows: Φ denotes fluence, *D* denotes dose, and *P* denotes passing rate. The subscript *c* represents quantities derived from calculation, while the subscript *m* represents quantities obtained from measurement. The superscript ′ indicates an error‐introduced plan (with the quantities pertaining to the original plan not including a superscript). $P_{I(\vec{r})}$ describes the passing rates of the intensity difference test and *P*_γ_ describes the passing rates of gamma analysis.

In our correlation study, dose was inferred from both dose calculation within the TPS and measurement with ArcCHECK. Calculated dose from the TPS provides 3D dose volumes thus allowing for QA in 3D, while the measurement‐based QA could only be in 2D and is relatively sensitive to setup errors. After delivery, the measured 2D dose volumes, denoted by *D*_*m*_ and $D_{m}^{\prime }$, respectively, are exported from the ArcCHECK software for evaluations. The measured doses $D_{m}^{\prime }$ from each error‐introduced plan are compared with the planned calculated dose from TPS (Pinnacle), i.e., *D*_*c*_.

For the passing rate results, we evaluate differences between: (1) calculated 3D fluences *Φ*_*c*_ and $\Phi_{c}^{\prime }$; (2) calculated 3D planned doses *D*_*c*_ and $D_{c}^{\prime }$; and (3) measured doses in phantom $D_{m}^{\prime }$ and calculated doses *D*_*c*_. 2% and 2%, 2 mm criteria are used in the intensity difference tests and gamma analysis for evaluating differences in (1) and (2), while 3% and 3%, 3 mm are chosen for evaluating differences in (3). The 2% and 2%, 2 mm criteria for (1) and (2) were selected empirically (similarly to the criteria choice for (3), as discussed previously). Furthermore, for (1) and (2), machine systemic errors, e.g., setup errors, are not included. Therefore, tighter constraints with 2%, rather than the 3% difference test and 3%, 3 mm gamma analysis are applied in the first two cases (1) and (2).

Finally, we obtain three groups of resultant passing rates with each group consisting of two test results from both evaluation methods, denoted by $P_{\Phi ,I(\vec{r})}$ and *P*_*Φ,γ*_, $P_{DC,I(\vec{r})}$ and *P*_*DC*,γ_, and $P_{Dm,I(\vec{r})}$ and *P*_*DC*,γ_. Five passing rates for each plan from five types of errors are obtained for these three result groups. Both Pearson's and Spearman's correlation coefficients are used to investigate the relationships between groups of passing rates. In particular, *r*‐values (Pearson's correlation coefficient) and *ρ*‐value (Spearman's correlation coefficients) are calculated to measure the extent to which two variables (e.g., $P_{\Phi ,I(\vec{r})}$ and $P_{DC,I(\vec{r})}$) tend to change together, including both the strength and the direction of the correlation.

## 3 Results

### 3.1 Clinical results

This reported 3DFC QA method has also been applied to QA at the authors’ institution for IMRT and VMAT treatments for the past 4 + yr. For pre‐treatment patient‐specific QA prior to 2014, a combination of ion chamber measurements (two points in a customized cubic solid water phantom), MapCheck QA (per beam at a fixed gantry angle of 0°), and fluence QA (using the log files acquired in delivery for ion chamber measurements) were applied. Since 2014, a combination of MatriXX QA (planar composite dose measurement of all beams in the coronal plan at isocenter, measured in the standard MatriXX iso‐cube water phantom) and fluence QA (log files acquired in the MatriXX QA delivery) have been used, with the MatriXX QA replacing both ion chamber and per‐beam MapCheck measurements. 2DFC QA, which was developed prior to the clinical implementation of VMAT, is applied to all IMRT plans. 3DFC QA, which was developed particularly for VMAT delivery verification, is applied to VMAT beams. 3DFC QA is currently not applied to IMRT plans, not because of technical limitations but rather for the sake of continuity in our institution's patient‐specific plan QA paradigm. 2D and 3D fluence‐based QA are implemented together with beam parameter checks in a fully automated treatment delivery verification program that is scheduled to run every morning to automatically check all treatment deliveries of the previous day and to alert physicists of any treatment delivery issues.

**Figure 3:**
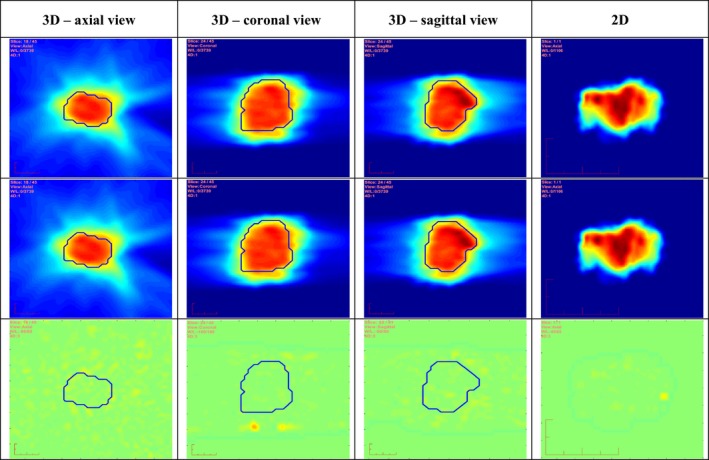
Results of 3D and 2D fluences from a four‐arc lung VMAT plan. Top row is from the DICOM plan. Middle row is from the log file. Bottom row is obtained by calculating the corresponding fluence differences. The PTV contours in the respective 3D orthogonal views are overlaid on the 3D fluences.

From 2014 to date, 139 VMAT treatments were verified using the reported 3DFC QA method (130 lung cancer patients, 5 heart cancer patients, 3 brain cancer patients, and 1 spine patient). Screenshots of this QA software and associated QA reports are illustrated in (Fig. 3) for one lung patient treated in 2015.

The reported 3DFC QA method was implemented in MATLAB 2012a (Mathworks, Natick, MA, USA). The computational accuracy and speed were tested for different choices of parameters. Voxel sizes and angular resolutions of 3 × 3 × 3 cm^3^ and 1° were ultimately chosen for 3D fluence calculations of both treatment plans and log records in order to obtain satisfactory spatial resolution and computation speed. Computation time ranges between 10 and 30 seconds per patient, with the speed ultimately depending on the number of VMAT beams and the size of the treatment target.

### 3.2 Lung plan

As lung patients are the most common VMAT‐treated patients in our clinic, we present one example of the delivery QA results including both the 3D and 2D fluences, using a four‐arc right lung VMAT plan with a total of 4328 MU. Figure 3 shows both the calculated 3D and 2D fluences integrated over the four arcs on the first two rows, including results obtained from both the DICOM plan and the log file, respectively. The corresponding calculated fluence map differences are shown on the third row. The 3D fluence volumes with PTV contours drawn are illustrated in axial, coronal, and sagittal views in the first three columns, respectively, while the 2D fluence (computed by forcing gantry angle = 0) is illustrated in the fourth column. Gantry angle, collimator, jaw, and MLC positions were all within allowed clinical tolerances during actual delivery. 3D fluence errors are 1.3 ± 1.1 MU and the maximal fluence error is 10.1 MU (0% failing rates for both criteria are found). As can be seen, there are only minimal fluence differences between the plan and delivery logs. More importantly, 3D fluence maps provide a significantly improved visualization in multiple orthogonal views on the PTV than the 2D map which cannot be associated with the PTV 3D shape.

### 3.3 Simulated delivery error results and analysis

As described in Section 2.5, we first simulated different types of machine errors per control point within normal machine tolerances. A total number of 10 patients with five lung (4‐arc) and five heart (3‐arc) VMAT plans were tested for simulated delivery errors. Results of averaged (mean value) failing rates for the 3% intensity fluence difference test and 3%, 3 mm gamma analysis, denoted by $F_{I(\vec{r}),3\%}$ and *F*_*γ*,3%,3mm_ respectively, are presented in (Table 2). For instance, using the 3DFC method, random gantry angle errors up to 1° could cause mean values of 7.4% and 6.2% of voxels to fail the 3% intensity difference test and 3%, 3 mm gamma analysis, respectively. In contrast, these simulated gantry angle errors were never detected by 2D fluence calculations because gantry angles are not used. Based on these results shown in Table 2, we may therefore conclude that the 3DFC QA method is more sensitive in detecting gantry angle errors, MU errors, jaw position errors, and collimator rotation errors than 2D fluence method.

**Table 2:** QA results with simulated delivery errors

Simulated errors	3D Fluence		2D Fluence		2D Measurement	
	$F_{I(\vec{r}),3\%}$	*F*_γ,3%,3mm_	$F_{I(\vec{r}),3\%}$	*F*_γ,3%,3mm_	$F_{I(\vec{r}),3\%}$	*F*_γ,3%,3mm_
**Errors within machine tolerance**						
Gantry	7.4%	6.2%	0%	0%	2.3%	1.4%
MU	1.2%	0.5%	0%	0%	0.7%	0%
Jaw	2.7%	1.6%	0.2%	0%	2.5%	0.2%
Collimator	1.6%	0.6%	0.3%	0%	0%	0%
MLC	14.3%	6.2%	21.1%	11.0%	13.5%	5.9%
**Errors outside machine tolerance**						
Gantry	14.1%	10.3%	3.3%	2.7%	11.5%	4.6%
MU	51.5%	37.7%	43.3%	24.2%	47.1%	36.9%
Jaw	11.6%	4.2%	4.8%	2.3%	5.2%	2.6%
Collimator	12.5%	7.3%	4.1%	9.3%	10.4%	3.5%
MLC	26.3%	9.3%	38.9%	24.7%	22.1%	5.9%

As also summarized in Table 2, we tested both algorithms upon adding simulated 1‐2 mm random MLC errors. We only adjusted the position of leaves that actually contribute to fluence during delivery. As one can see, both 3D and 2D methods are very sensitive to MLC position errors. However, the results suggest that the 3DFC QA method is less sensitive to MLC positional errors than the 2D method. This might be due to that MLC positional errors only affect the beam fluence at the edges but not inside of beam portals. ArcCHECK measurements were performed on these unmodified VMAT test plans. For each plan, the measured dose will be compared with 10 calculated dose files from error‐introduced plans (see Section 2.5). Results presented in (Table 2) show that 3DFC QA is more sensitive to these simulated machine errors than the conventional measurement‐based QA.

**Figure 4:**
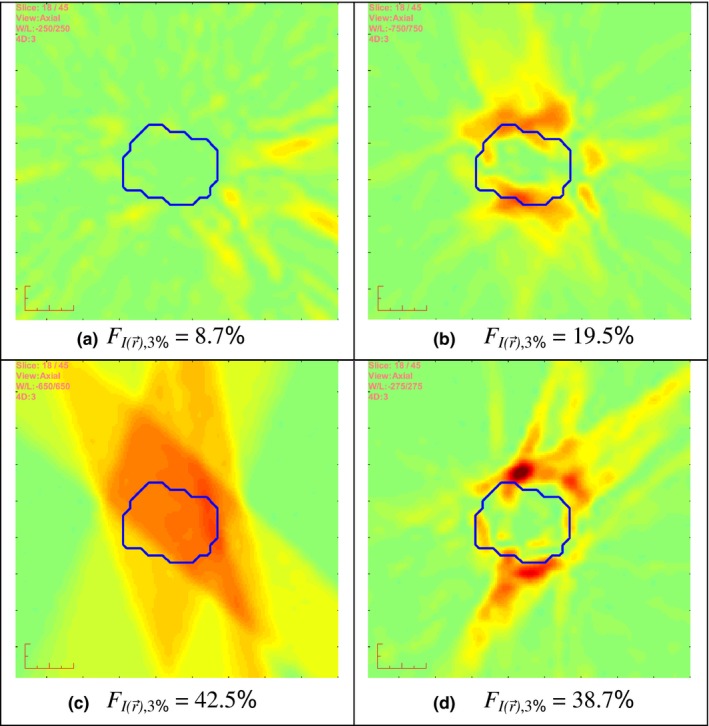
The axial views of fluence differences generating by simulated errors: (a) with fixed 1° gantry angle errors; (b) with fixed 2 mm shifting MLC leaf position errors; (c) 90% of the plan is interrupted during delivery; and (d) an incorrect version of the plan is delivered.

Furthermore, simulated errors outside their corresponding normal tolerances are tested. Significantly greater errors are found in this case which demonstrates both the conclusion on 3DFC method achieving better sensitivity than the 2DFC. Two examples of the calculated 3D fluence differences in axial views are illustrated in (Fig. 4), where (a) is generated using fixed 1° gantry angle error in one lung plan, and (b) is generated with fixed 2 mm MLC position errors in the same lung plan. These artificial errors can be visually seen in the 3D fluence difference map, which also suggest the feasibility of our method.

Figure 4 also illustrates two 3D fluence difference maps generated by simulating two common clinical events: interruptive delivery with 10% of the control points left out during treatment, and delivering the wrong version of the plan (of another lung patient used).

**Figure 5:**
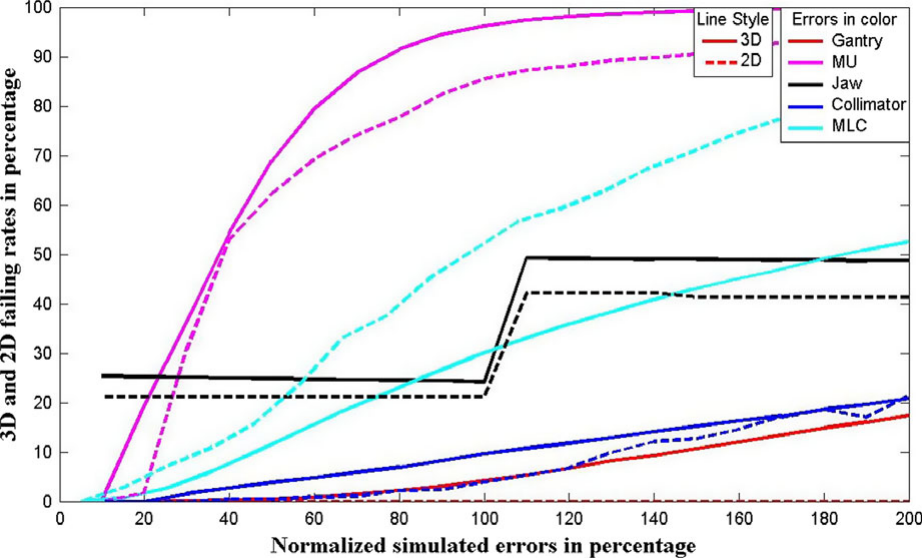
Correlations between the simulated errors and the 3% fluence differences test failing rates for various types of errors using both 3DFC QA in solid lines and 2D fluence QA in dashed lines.

In order to better understand the relationship between the delivery errors and the performance of the 3DFC QA method, different values of artificial errors for each error type are added into each control point and the corresponding average (mean value) failing rates of the 3D fluence difference test (3%) are obtained for these 10 VMAT plans. Figure 5 shows plots of the correlations between the normalized simulated errors and the failing rates both in percentages for all the error types examined in this paper, while results of the reported 3DFC QA (in solid lines) are compared with those of the 2D fluence‐based QA (in dashed lines). The generated errors are normalized to the corresponding tolerance in percentage, for instance, gantry angle errors are scaled to 100% at 1°. As can be seen, failing rates start to climb much more quickly when the errors lie outside their normal machine tolerances. It again demonstrates the conclusion from Table 2 that 3DFC algorithm is more sensitive to MU, jaw position, and collimator rotation errors than 2DFC, while 2DFC cannot catch gantry rotation errors.

### 3.4 Correlation study results on fluence vs. dose and analysis

In the correlation study, 10 IMRT plans and 10 VMAT plans with five different types of errors were used. Table 3 presents the computed correlations between $P_{\Phi ,I(\vec{r})}$ and $P_{DC,I(\vec{r})}$, and between *P*_*Φ*,*γ*_ and *P*_*DC*,*γ*_. Table 4 presents the computed correlation between $P_{\Phi ,I(\vec{r})}$ and $P_{Dm,I(\vec{r})}$, and between *P*_*Φ*,*γ*_ and *P*_*Dm*,*γ*_. In both tables, the Spearman's correlation coefficient *ρ*‐value is significantly closer to +1 than the Pearson's correlation coefficient *r*‐value. The *p*‐values for all errors types are smaller than the significance level 5%, which suggest the statistically significant correlation. These results show that the resultant passing rates indicated by 3DFC are correlated with the passing rates specified by dose mapping, obtained from either calculation or measurement.

**Table 3:** Results of correlation coefficients between *P*_*Φ*_ and *P*_*Dc*_ from all the error types

Coefficients	Gantry	MU	Jaw	Collimator	MLC
$P_{\Phi ,I(\vec{r})}$ **and** $P_{Dc,I(\vec{r})}$ **(2% intensity difference)**					
Pearson (r)	0.5631	0.5489	0.7025	0.8575	0.5297
Spearman (*ρ*)	0.9267	0.9183	0.9226	0.9515	0.6754
Spearman (p)	0.0427	0.0242	0.0475	0	0.0305
*P*_*Φ*,*γ*_ **and ** *P*_*Dc*,*γ*_ **(2%, 2mm Gamma analysis)**					
Pearson (r)	0.6125	0.5987	0.7871	0.8824	0.5011
Spearman (*ρ*)	0.9334	0.9423	0.9498	0.9817	0.7042
Spearman (p)	0.0375	0.0197	0.0420	0	0.0421

**Table 4:** Results of correlation coefficients between *P*_*Φ*_ and *P*_*Dm*_ from all the error types

Coefficients	Gantry	MU	Jaw	Collimator	MLC
$P_{\Phi ,I(\vec{r})}$ **and** $P_{Dm,I(\vec{r})}$ **(3% intensity difference)**					
Pearson (r)	0.4121	0.6514	0.5248	0.8554	0.4336
Spearman (*ρ*)	0.9701	0.8997	0.9015	0.9810	0.7853
Spearman (p)	0.0232	0.0399	0.0425	0.0315	0.0652
*P*_*Φ*,*γ*_ **and ** *P*_*Dm*,*γ*_ **(3%, 3mm Gamma analysis)**					
Pearson (r)	0.5771	0.5397	0.6541	0.7981	0.5026
Spearman (*ρ*)	0.8653	0.9012	0.9520	0.9805	0.7916
Spearman (p)	0.04196	0.0356	0.0492	0.0157	0.0694

## 4 Discussion

3DFC QA is useful to detect relatively common treatment delivery imperfections (e.g., imperfect deliveries due to the end effect for highly modulated IMRT beams that deliver low MUs, and treatment delivery interruptions). Although overall treatment delivery accuracy has been significantly improved by newer LINAC machines, e.g., Varian's TrueBeam, the reported 3DFC QA can serve as an additional safeguard for error checking. Additionally, the reported method can continue to provide QA for older, more error‐prone machines.

Comparing to the previous treatment delivery log‐based QA methods, 3DFC QA is capable of presenting the QA results and the computed beam fluences in 3D geometry and therefore allows users to easier interpret QA results in terms of the patient 3D anatomy and the PTV target. In comparison to 2DFC (using composite fluence maps and ignoring gantry rotation), 3DFC QA can detect important treatment delivery errors, such as gantry angle errors, and is more sensitive to MU, jaw position, and collimator rotation errors. For the case of MLC errors, even though the 3D method is less sensitive than the 2D method, the sensitivity is sufficient for the general VMAT delivery verification purpose. In comparison to other methods that only check beam parameters in delivery log files, e.g., FractionCheck (Mobius Medical System, Houston, TX, USA), 3DFC compares 3D fluences derived from log files with those specified by the treatment plans, thus enabling detection of frequently occurring treatment plan data transfer errors (incorrect plan version, incorrect version of the individual beams.^11^

The described 3DFC method is not designed to catch most errors in treatment planning system, e.g., imperfect beam modeling. Its primary application is instead to catch certain rare errors such as the junctions of closed MLC leaf pairs left inside the beam field defined by the X and Y jaws in Pinnacle step‐and‐shoot IMRT plans.^11^ Compared to measurement‐based QA, the results of 3DFC QA are less independent because the beam parameters in the deliveries were measured by the treatment machines instead by independent measurement device. The beam output and the beam profile are not directly measured, either. The accuracy of the beam parameters provided in the machine log files must be independently verified through routine machine QA in order to be considered reliable. In fact, in one reported incident MLC positions recorded in the log file were shown to be inconsistent with observed, true positions.^37^ Therefore, concerns and debates continue on the merits of log‐file based QA.^5^ For these reasons, the reported 3DFC method is currently used as a complementary tool to measurement‐based QA for pre‐treatment IMRT and VMAT patient‐specific QA in our clinic. Once confidence has been established via pre‐treatment QA derived from calculation and measurement, 3DFC is used to verify the subsequent patient treatment deliveries.

3DFC is also not designed to replace a full dose calculation, but as an alternative approach as a delivery QA with enhanced visualization and error sensitivity, focusing directly on checking machine parameters. Comparing to full 3D dose calculation methods, 3DFC ignores many important physical effects including phantom scattering and attenuation. However, 3DFC is simpler and could be potentially much faster. The current computation speed, 3 to 20 seconds per VMAT beam, accomplished with MATLAB programs could also be significantly improved when 3DFC is reimplemented in C/C++ or GPU programs. It might be worth to note that the computation speed of 3DFC is sufficient for clinical use without GPU acceleration. This allows the reported QA method to be more clinically deployable without the need of relatively expensive GPU hardware. As shown in (Section 3.4), 3DFC passing rates are correlated with passing rates inferred from measurement‐based QA. The correlation results could be interpreted as a monotonic trend observed for the two obtained passing rates, i.e., fluence vs. dose, where higher fluence errors indicates higher dose errors, and lower fluence errors indicates lower dose errors. Given the same error type and threshold, the 20 observations (i.e., passing rates) obtained for both QA methods can be considered as two ranked variables in the Spearman's correlation, which is proven to be much stronger than Pearson's linear relationship.

Even though the reported 3DFC method digitally reconstructs 3D fluence from the treatment plan and treatment delivery logs, 3D fluence can be also reconstructed from measured 2D fluence using 2D diode arrays mounted on the gantry and rotate together with the gantry by:

(8) $$I\left(\vec{r}\right)=\Delta t\sum_{k}G_{k}\left(\vec{r}\left(t\right)\right)\times \frac{SAD^{2}}{\left(\vec{r}-{\vec{s}}_{k}\right)}$$

where *G* is a frame of the measured fluence map movie, *Δt* is the measurement repetition period. Beam MU is not in this equation because it is reflected by the intensity of measured beam fluence. Gantry angles must be simultaneously measured.

## 5 Conclusion

An efficient method, 3DFC, has been developed to calculate 3D fluence volumes using the beam parameters from both DICOM plan files and machine delivery log files for verifying both IMRT and VMAT treatment deliveries. This method is designed to work complementarily to other QA procedures including dose recalculations and phantom‐based measurements in order to provide a quick and easy measurement of beam delivery fidelity and better visual presentation of delivery errors in 3D. The reported method could be useful in catching both treatment plan data transfer errors and treatment delivery problems.

## Conflict of Interest

Authors have no conflict of interest.
